# Mitochondrial Protein PGAM5 Emerges as a New Regulator in Neurological Diseases

**DOI:** 10.3389/fnmol.2021.730604

**Published:** 2021-09-23

**Authors:** Min-Zong Liang, Ting-Ling Ke, Linyi Chen

**Affiliations:** ^1^Institute of Molecular Medicine, National Tsing Hua University, Hsinchu, Taiwan; ^2^Department of Medical Science, National Tsing Hua University, Hsinchu, Taiwan

**Keywords:** PGAM5, mitochondrial homeostasis, mitochondrial dynamics, neurological diseases, mitophagy

## Abstract

As mitochondrial dysfunction has increasingly been implicated in neurological diseases, much of the investigation focuses on the response of the mitochondria. It appears that mitochondria can respond to external stimuli speedy fast, in seconds. Understanding how mitochondria sense the signal and communicate with cytosolic pathways are keys to understand mitochondrial regulation in diseases or in response to trauma. It was not until recently that a novel mitochondrial protein, phosphoglycerate mutase family member 5 (PGAM5) has emerged to be a new regulator of mitochondrial homeostasis. Although controversial results reveal beneficial as well as detrimental roles of PGAM5 in cancers, these findings also suggest PGAM5 may have diverse regulation on cellular physiology. Roles of PGAM5 in neuronal tissues remain to be uncovered. This review discusses current knowledge of PGAM5 in neurological diseases and provides future perspectives.

## Introduction

Brain is an energy-expensive organ, consuming about 20 percent of our total energy (Rolfe and Brown, 1997). Mitochondria synthesize ATP to meet the high energy demand of our brain and sustain cellular physiology. To meet the energy expenditure of neurons, mitochondria traffic between soma, dendrites and the long axons (Misgeld and Schwarz, 2017). The function and quality control of mitochondria are in part controlled through mitochondrial morphogenesis, fission and fusion, as well as through mitophagy and mitochondrial biogenesis. Several dynamin-related GTPases that regulate mitochondrial fission and fusion have been identified. Mitochondrial GTPase mitofusins, MFN1 and MFN2, mediate fusion between two mitochondrial outer membranes (MOM), followed by fusion of mitochondrial inner membrane (MIM) mediated by optic atrophy 1 (OPA1). Mitochondrial fusion increases oxidative capacity and mitigates stress by mixing proteins and lipids between mitochondria (Westermann, 2010; Youle and van der Bliek, 2012). Mitochondrial fission, on the other hand, is mediated by cytoplasmic GTPase dynamin-related protein 1 (DRP1). As yeast genetic study identified mitochondrial fission 1 (FIS1) being required for DRP1-mediated fission (Mozdy et al., 2000), this role of FIS1 has been controversial compared to metazoans and mammals (Otera et al., 2010; Shen et al., 2014). In response to stress, DRP1 is recruited to mitochondria, interacts with mitochondrial fission factor (MFF), and enters into a fission complex containing FIS1 at endoplasmic reticulum-mitochondria interface. While FIS1 mutations had minimal effect on fission, it acted with MFF during subsequent degradation (Shen et al., 2014). Through modulating expressions of MFF and FIS1, Otera et al. (2010) showed that MFF-DRP1 governed mitochondrial fission but not FIS1. Thus, whether MFF or FIS1 is the acceptor of DRP1 is likely context-dependent. Oligomers of DRP1 assemble on MOM as spiral shape to constrict MOM and MIM and divide mitochondria into daughter organelles at the expense of GTP hydrolysis (Youle and van der Bliek, 2012; Ni et al., 2015; Sedlackova and Korolchuk, 2019). While mitochondrial fission facilitated the separation of damaged subdomains from the healthy part, fission was shown not necessarily required for all mitophagy (Mendl et al., 2011; Burman et al., 2017). Autophagic elimination of mitochondria, termed mitophagy, is mediated by phosphatase and tensin homolog-induced putative kinase protein 1 (PINK1) and Parkin. PINK1 is imported to MIM and degraded by rhomboid protease presenilin-associated rhomboid-like (PARL) in healthy mitochondria. Upon mitochondrial damage, the transport and degradation of PINK1 are inhibited, allowing accumulation of PINK1 on MOM. Accumulated PINK1 recruits the E3 ubiquitin ligase Parkin from cytosol to damaged mitochondria. Parkin ubiquitinates MOM proteins and induces autophagic degradation of mitochondria (Youle and Narendra, 2011; Youle and van der Bliek, 2012). Mitochondrial fission is also involved in mitochondrial biogenesis, a process that generates new mitochondria to preserve population and function of mitochondria. During mitochondrial biogenesis, nuclear genome and mitochondrial DNA (mtDNA) encode mitochondrial proteins and new mitochondria are generated *via* mitochondrial fission (Palikaras and Tavernarakis, 2014; Popov, 2020). Nuclear respiratory factors, NRF1 and NRF2, regulate the expression of mitochondrial respiratory chain complexes and mitochondrial enzymes through binding to specific enhancer antioxidant response elements (AREs) of target gene (Johnson et al., 2008). The function of NRF1 and NRF2 are in turn controlled by the major regulator, coactivator PGC1-α (Bouchez and Devin, 2019; Gureev et al., 2019). PGC1-α facilitates the nuclear translocation of NRF2 *via* p38 MAPK-mediated GSK3β inactivation (Choi et al., 2017), which has been shown to phosphorylate Fyn kinase and enhance nuclear export of NRF2 (Jain and Jaiswal, 2007). Nuclear NRF2 binds to AREs at the promoters of *Pgc1-α* and *Nrf1* genes, increasing expressions of NRF1 and PGC1-α (Baldelli et al., 2013; Hong and Lee, 2018). Furthermore, NRF1 and NRF2 induce expression of mitochondrial transcription factor TFAM, which, in turn, enhance transcription and replication of mtDNA (Virbasius and Scarpulla, 1994).

Unbalanced mitochondrial homeostasis has been implicated in several neurological diseases (Misgeld and Schwarz, 2017; Chang et al., 2019). In Alzheimer’s disease (AD), DRP1 and FIS1 levels were upregulated. In the presence of excessive β-amyloid, DRP1 nitrosylation could lead to mitochondrial fission. In contrast, mitochondrial fusion proteins, MFN1, MFN2 and OPA1, were decreased in the brain of AD patients. Reduction of DRP1 maintained mitochondrial function, reduced β-amyloid and enhanced synaptic activity in the AD mouse model (Cho et al., 2009; Manczak et al., 2011; Gollihue and Rabchevsky, 2017). Similarly, overexpression of Parkin has been shown to reduce β-amyloid load, remove damaged mitochondria and rescue synaptic plasticity in AD models (Hong et al., 2014; Wang et al., 2020). Abnormal mitophagy was also reported in *in vitro* and *in vivo* models of Parkinson’s disease (PD; Chinta et al., 2010; Cherra et al., 2013; Chu et al., 2013; Tresse et al., 2021). For patients with glioma, ionizing radiation is a choice of treatment after brain surgery. However, the tissue surrounding the brain lesion inevitably receives a low dose of radiation. To examine how hippocampal neurons respond to the low level stimulus, Chien et al. (2015) demonstrated that increased MFN2 and reduced DRP1 activity promoted mitochondrial fusion as an adaptive mechanism. On the other hand, mitochondrial fission occurred quite early after ischemic stroke-induced neuronal damage. Inhibition of DRP1 or overexpression of MFN1 prevented mitochondrial fission and apoptosis induced by ischemia injury (Barsoum et al., 2006; Grohm et al., 2012; Wang et al., 2014). These studies demonstrate that removing damaged mitochondria through fission is essential in maintaining mitochondrial homeostasis and health of the nervous systems. A novel phosphoglycerate mutase (PGAM) family member, PGAM5, has emerged to be a regulator of mitochondrial homeostasis. PGAM5 belongs to PGAM family but functions as a Ser/Thr protein phosphatase in the mitochondria (Takeda et al., 2009; Wang et al., 2012). The N-terminal 35 amino acids of PGAM5, containing a transmembrane domain, target PGAM5 to mitochondria (Lo and Hannink, 2008). PGAM5 formed stable dimers through its C-termini (Chaikuad et al., 2017) while its N-terminal contains a conserved WDXNWD motif, required for PGAM5 multimerization. Based on the structural analyses, function of PGAM5 is regulated by multimerization, either as dimers or dodecameric forms (Chaikuad et al., 2017; Ruiz et al., 2019). Mutation of WDXNWD motif disrupts PGAM5 dodecamer and reduces phosphatase activity of PGAM5 (Wilkins et al., 2014; Ruiz et al., 2019). PGAM5 dimers, on the other hand, dephosphorylate anti-apoptotic protein BCL-xL to prevent apoptosis. Under oxidative stress, PGAM5 stabilized PINK1 on mitochondria and thus promoted PINK1/Parkin-mediated mitophagy (Sekine et al., 2012; Lu et al., 2014; Yan et al., 2020). Another line of evidence showed that PGAM5 also engaged in PINK1-independent mitophagy by dephosphorylating mitophagy receptor FUNDC1 (Chen et al., 2014; Ma et al., 2019). In this respect, PGAM5 formed dodecamer and dephosphorylated mitophagy receptor FUNDC1 to enhance mitophagy (Ma et al., 2019).

When mitochondrial membrane potential is compromised, PGAM5 is cleaved at transmembrane domain by PARL and released its C-terminal phosphatase domain to cytosol. The release of cleaved PGAM5 from mitochondria allows PGAM5 to interact with and dephosphorylate its candidate substrates in cytosol (Sekine et al., 2012; Bernkopf et al., 2018), such as β-catenin to activate WNT canonical signaling in cancer cells (Bernkopf et al., 2018). Canonical WNT/β-catenin signaling induced by WNT3A promoted mitochondrial biogenesis in C2C12 myoblast cells (Yoon et al., 2010). Thus, a role of PGAM5 in mitochondrial biogenesis is speculated. Nonetheless, exactly how PGAM5 may regulate mitochondrial homeostasis in neurological diseases remains largely unknown. In this review, we discuss the potential role of PGAM5 in mitochondrial homeostasis and neurological diseases.

## PGAM5 Dephosphorylates DRP1 to Promote Mitochondrial Fission

**Figure 1:**
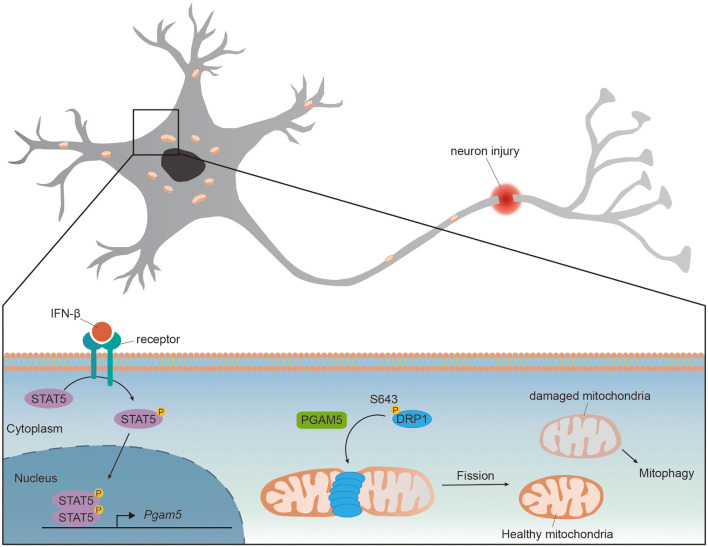
PGAM5 dephosphorylates DRP1 to promote mitochondrial fission. The expression level of PGAM5 is upregulated in neurons upon injury or IFN-β treatment. IFN-β binds to IFNAR receptors and activates transcription factor STAT5. Activated STAT5 translocates to nucleus and enhances expression of *Pgam5*. The upregulated PGAM5 dephosphorylates Ser643 of DRP1 and recruits DRP1 to mitochondria, promoting mitochondrial fission and the elimination of damaged part of mitochondria.

Wang et al. first demonstrated that PGAM5 regulated mitochondrial fission by dephosphorylating DRP1 in HeLa cells. The GTPase activity of DRP1 is regulated by phosphorylation at Ser637 [pDRP1(S637)] to inhibit DRP1 activity and repress mitochondrial fission (Chang and Blackstone, 2007). Upon necrosis induction, PGAM5 dephosphorylated pDRP1(S637) and enhanced DRP1 activity whereas knockdown of PGAM5 prevented mitochondrial fission and necrosis in HeLa cells (Wang et al., 2012). Deletion of PGAM5 promoted mitochondrial elongation in HeLa and retinal pigment epithelial cells, indicating that PGAM5 also regulated mitochondrial fission in healthy cells (Sugo et al., 2018; Yu et al., 2020). Upon traumatic brain injury (TBI), the expressions of PGAM5 and DRP1 were increased in mice brain whereas pDRP1(Ser643) [human DRP1(Ser637)] was reduced, suggesting an increased PGAM5 activating DRP1 *via* its phosphatase activity. Knockdown of PGAM5 attenuated DRP1 activity, increased mitochondrial membrane potential and restored ATP production in injured primary cortical neurons (Chen et al., 2021). These results support the requirement of PGAM5 for mitochondrial fission and function. A recent study demonstrated that cytokine interferon-β (IFN-β) enhanced expression of PGAM5 and activated mitochondrial fission in PD models (Tresse et al., 2021). Large aggregates of mitochondria were found in IFN-β knockout (*Ifnb*^−/−^) mice brain but not in the wild type. The accumulated mitochondria in *Ifnb*^−/−^ mice were accompanied with impaired mitochondrial function and resulted in spontaneous neurodegeneration and PD-like dementia (Ejlerskov et al., 2015; Tresse et al., 2021). Despite the lower PGAM5 protein and mRNA levels in *Ifnb*^−/−^ neurons compared to those in the wild type, in the presence of intact IFN-β receptor, the addition of recombinant IFN-β increased PGAM5 level to a similar level in both wild type and *Ifnb*^−/−^ neurons. Mechanistically, brain IFN-β maintained mitochondrial homeostasis and prevented neurodegeneration *via* STAT5/PGAM5 pathway in 6-hydroxydopamine-induced PD model or genetic PD model (Tresse et al., 2021). Since the expression of IFN-β was increased in mice TBI model (Barrett et al., 2020), it is possible that the upregulated PGAM5 upon TBI is induced by IFN-β (Figure 1).

## PGAM5 Enhances PINK1/Parkin-Mediated Mitophagy

**Figure 2:**
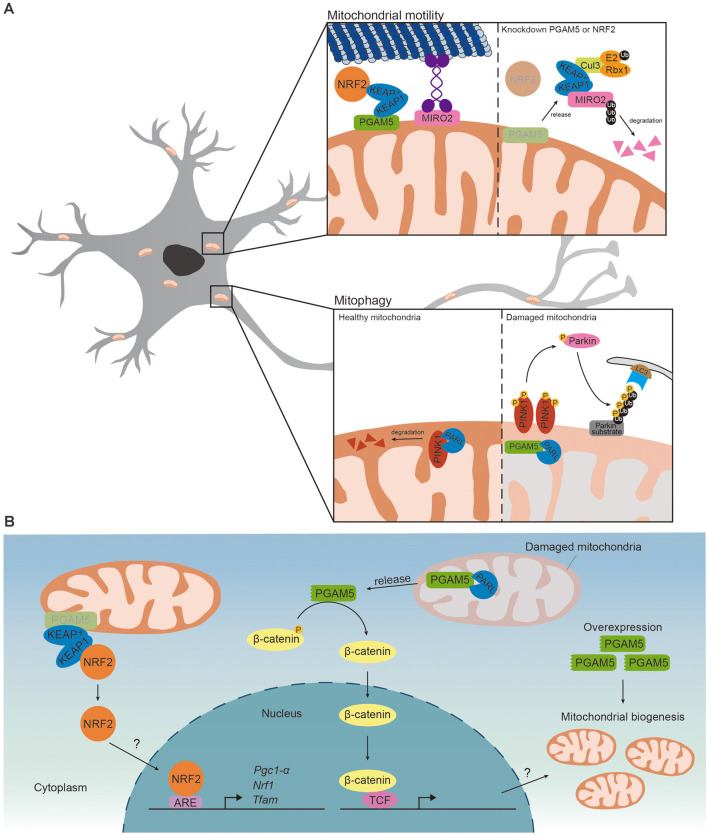
PGAM5 regulates mitochondrial motility and mitophagy. **(A)** (Upper) In the presence of PGAM5-KEAP1-NRF2 complex on the MOM, Miro2 would link kinesin to the MOM and regulate mitochondrial retrograde trafficking induced by proteasome inhibition. While knockdown of PGAM5 or NRF2, KEAP1 is released from the complex and free KEAP1 couples with Cul3 forming ubiquitin complex and result in degradation of Miro2 resulting in the inhibition of mitochondrial retrograde trafficking. (Lower) PINK1 is transported to MIM and degraded by PARL in healthy mitochondria. In damaged mitochondria, PARL cleaves PGAM5, instead of PINK1, which stabilizes PINK1 on MOM. Accumulation of PINK1 would recruit Parkin to ubiquitylate Parkin substrates on MOM. The autophagosome with LC3 and autophagy receptor would then be recruited to trigger Parkin-mediated mitophagy. **(B)** (Left) PGAM5-KEAP1-NRF2 complex restricts NRF2 to mitochondria and represses NRF2-dependent transcription. Upon knockdown of PGAM5, NRF2 translocates to the nucleus and activates NRF2-dependent transcription. But whether PGAM5 inhibits mitochondrial biogenesis *via* repressing NRF2-dependent transcription is unclear. (Right) When mitochondria sense stress signal, PGAM5 is cleaved by PARL and released to cytoplasm. Cleaved PGAM5 dephosphorylates β-catenin and activates β-catenin-dependent transcriptions. Overexpressing cleaved PGAM5 promotes mitochondrial biogenesis. However, whether PGAM5 promotes mitochondrial biogenesis *via* β-catenin-dependent transcriptions remains unknown.

Both PGAM5 and PINK1 are cleaved by PARL, thus it is not surprising to find that PGAM5 competes with PINK1 for PARL (Sekine et al., 2012). PINK1 is transported to MIM and degraded by PARL in healthy mitochondria. Upon loss of membrane potential, PARL cleaved PGAM5, instead of PINK1, stabilizing PINK1 on MOM and inducing PINK1/Parkin-mediated mitophagy (Figure 2A; Sekine et al., 2012; Yan et al., 2020). The regulatory role of PGAM5 in PINK1/Parkin-mediated mitophagy has been reported in SH-SY5Y neuroblastoma cells (Park et al., 2018). Carbonyl cyanide m-chlorophenyl hydrazone (CCCP) treatment induced loss of mitochondrial membrane potential and increased expression of PGAM5, PINK1 and Parkin. Upregulated PINK1 and Parkin activated PINK1/Parkin-mediated mitophagy to eliminate defective mitochondria. Knockdown of PGAM5 repressed CCCP-induced expressions of PINK1 and Parkin and thus mitophagy, suggesting that PGAM5 is required for removing damaged mitochondria *via* mitophagy (Park et al., 2018). Sugawara et al. (2020) recently reported that knockout of PGAM5 enhanced oxygen consumption rate in adipocytes. As mitochondrial mass and oxygen consumption rate are increased in the adipocytes of Parkin-deficient mice, it is possible that PGAM5 represses oxygen consumption through Parkin-dependent mitophagy (Lu et al., 2018). Disrupted mitophagy has been reported in PD patients and several PD models (Liu et al., 2019). It is possible that PGAM5 regulates mitophagy to prevent neurodegeneration in PD. PGAM5 deficiency disabled mitophagy and led to a PD-like phenotype *in vivo* (Lu et al., 2014). The level of dopamine and the survival of dopaminergic neurons were reduced in aged PGAM5-knockout mice, indicating that PGAM5 prevented neurodegeneration. Along the same line, PGAM5-knockout mice exhibited impaired motor function similar to those seen in PD patients (Lu et al., 2014). On the other aspect, PGAM5 mediated mitophagy to promote neuroprotection against ischemic reperfusion injury. The infarct size and neurological deficits in limb movement were increased in PGAM5 knockout mice upon middle cerebral artery occlusion injury (Lu et al., 2016). Thus, PGAM5 functions to regulate mitophagy to prevent neurodegeneration and promote neuroprotection. Based on these studies, the relative level of PGAM5 is likely to reflect its functional demand in a specific physiological context under a specific phase of the disease progression.

## Potential Role of PGAM5 in Mitochondrial Biogenesis

WNT/β-catenin signaling has been reported to regulate mitochondrial biogenesis in C2C12 myoblast cells by WNT3A-induced canonical WNT signaling (Yoon et al., 2010). In the absence of WNT ligands, β-catenin destruction complex phosphorylates β-catenin, leading to degradation of β-catenin. WNT ligand activates WNT/β-catenin signaling *via* inhibiting β-catenin destruction complex and stabilizing β-catenin in cytoplasm. Stabilized β-catenin translocates into the nucleus and enhances β-catenin-dependent transcription (Komiya and Habas, 2008). WNT/β-catenin signaling increased transcription of oxidative phosphorylation complexes and mtDNA copy number in C2C12 cells (Yoon et al., 2010). PGAM5 activated WNT/β-catenin signaling *via* dephosphorylation and stabilization of β-catenin in HEK293T and cancer cells (Bernkopf et al., 2018). When PGAM5 was cleaved by PARL and released to cytosol, it bound to and dephosphorylated β-catenin, leading to stabilization of β-catenin. Similarly, overexpression of cleaved PGAM5 increased mitochondrial mass and mtDNA copy number in C2C12 cells (Bernkopf et al., 2018). Nonetheless, it remains debatable whether cleaved PGAM5 enhances mitochondrial biogenesis *via* activating WNT/β-catenin signaling (Figure 2B). As the cleavage of PGAM5 is known to turn on WNT/β-catenin signaling, PGAM5 also antagonizes WNT/β-catenin signaling. PGAM5 does so through dephosphorylating disheveled, a positive regulator of WNT signaling, and thus inactivating WNT/β-catenin signaling. Knockdown of PGAM5 enhanced β-catenin-dependent transcription (Rauschenberger et al., 2017). While the current evidence do not explain themselves, it is possible that PGAM5-mediated mitochondrial biogenesis could be either WNT/β-catenin-dependent or -independent.

Another mechanism that PGAM5 mediates mitochondrial biogenesis is through NRF2. PGAM5 forms a ternary complex with Kelch-like ECH-associated protein 1 (KEAP1) dimer and NRF2. PGAM5 anchors the ternary complex onto mitochondria and restricts NRF2 to mitochondria. Release of NRF2 from the ternary complex is required for activation of NRF2-dependent gene expression. Consequently, PGAM5-KEAP1-NRF2 complex represses nuclear translocation of NRF2 and NRF2-dependent gene expression (Lo and Hannink, 2008). Additionally, PGAM5 might repress NRF2-dependent transcription *via* regulating export of NRF2 from nucleus. Xue et al. (2015) hypothesizes that PGAM5 dephosphorylates Fyn and activates Fyn-mediated nuclear export of NRF2 but more evidence is required to support their hypothesis. Knockdown of PGAM5 enhanced NRF2 nuclear translocation and NRF2-dependant transcription of anti-oxidant genes in macrophages, HeLa cells and microvascular endothelial cells (Lo and Hannink, 2008; Xue et al., 2015; Hos et al., 2017). While these results support the role of PGAM5 in limiting mitochondrial biogenesis *via* repressing NRF2, it remains to be determined whether nuclear translocation of NRF2 is facilitated by reduced PGAM5 (Figure 2B).

In contrast, PGAM5 facilitated hemin-induced mitochondrial biogenesis in hypoxia/reoxygenation (H/R) injury model (Hong and Lee, 2018). Hemin administration induced NRF2 nuclear translocation in a dose-dependent way (Nakaso et al., 2003). Hemin increased the protein levels of PGAM5, PGC1-α, NRF1 and TFAM in H/R cells. PGAM5 knockdown abolished the increase of PGC1-α, NRF1 and TFAM induced by hemin (Hong and Lee, 2018). These results suggest that hemin enhances mitochondrial biogenesis *via* upregulating PGAM5. Hemin induced both NRF2 nuclear translocation and protein level of PGAM5 (Nakaso et al., 2003; Hong and Lee, 2018), arguing against the PGAM5-mediated repression of NRF2 (Lo and Hannink, 2008). Given the obvious contradictory results, the interplay between PGAM5 and NRF2 during mitochondrial biogenesis will require additional investigation.

While regulation of PGAM5-NRF2 mediated mitochondrial biogenesis in neurological diseases had not been previously reported, a recent study revealed that interaction between PGAM5 and NRF2 resulted in blood–brain barrier disruption and neurological deficits in ischemic stroke (Gao et al., 2021). Treatment of a novel inhibitor of PGAM5, LFHP-1c, inhibited phosphatase activity of PGAM5, disrupted PGAM5-NRF2 interaction, and thus allowed nuclear translocation of NRF2. Consequently, NRF2-dependent expression of antioxidative genes reduced brain edema and improved neurological deficit induced by transient middle cerebral artery occlusion injury (Gao et al., 2021). The role of PGAM5-NRF2 axis in neurological diseases is at its primitive stage and will require further studies to coin its impact.

## PGAM5 Regulates Mitochondrial Transport Under Stress Conditions

Mitochondria buffer intracellular calcium during redox homeostasis and apoptosis (Jeong and Seol, 2008; Griffiths and Rutter, 2009). They themselves also serve as the source of intracellular reactive oxygen species (ROS; Lambert and Brand, 2009). Therefore, delicate regulation of mitochondrial function and distribution is crucial to optimize ATP production while minimizing excess ROS simultaneously. Cellular distribution of mitochondria is mediated by a complex trafficking on the microtubules. Bidirectional transport of mitochondria along the microtubule is primarily mediated by motor proteins, kinesin and dynein (Tanaka et al., 1998; Varadi et al., 2004; Pilling et al., 2006). Motor proteins anchor to mitochondria through the adapter complexes which consist of adaptor trafficking kinesin protein 1 and 2 and the small mitochondrial Rho GTPases Miro1 or Miro2 (Fransson et al., 2006; Russo et al., 2009; Koutsopoulos et al., 2010; van Spronsen et al., 2013).

Recent studies revealed a role of PGAM5 in mitochondrial transport under stress conditions. Knockdown of PGAM5 or NRF2 released KEAP1 from complex to interact with cullin-3, a scaffold protein for E3 ubiquitin ligase complex, and subsequently mediated degradation of Miro2, leading to inhibition of mitochondrial retrograde trafficking. Furthermore, the expression of Miro was reduced in the brain of NRF2 knockout mice (O’Mealey et al., 2017), revealing the possibility that PGAM5-KEAP1-NRF2 complex is required for mitochondrial transports in neurons (Figure 2A). Another study showed that infection of neurotropic herpes simplex viruses 1 altered mitochondrial motility in neurons *via* PGAM5 (Manivanh et al., 2020). Herpes simplex viruses 1-encoded neurovirulence protein, infected-cell protein 34.5 (ICP34.5), interacted with PGAM5 and reduced mitochondrial velocity in primary superior cervical ganglion neurons and trigeminal neurons. Disrupting interaction between ICP34.5 and PGAM5 restored the decreased mitochondrial velocity in neurons upon herpes simplex viruses 1 infection (Manivanh et al., 2020). However, ICP34.5/PGAM5 interaction did not affect activation of NRF2 and antioxidant response, suggesting that herpes simplex viruses 1 infection-mediated mitochondrial motility might be independent of PGAM5-KEAP1-NRF2 complex. Further studies are required to decipher exactly how PGAM5 regulates mitochondrial motility in neurons.

## Discussion and Future Perspective

PGAM5-mediated mitochondrial fission and mitophagy prevented neurodegeneration in PD and ischemic injury (Lu et al., 2014, 2016; Tresse et al., 2021) but the induction of PGAM5 by TBI promoted mitochondrial fission and impaired mitochondrial function (Chen et al., 2021). These findings implicate a disease-specific regulation of PGAM5 in neurological diseases. It is likely that manipulating PGAM5 level could improve the imbalance of mitochondrial homeostasis in neurological diseases. For instance, recombinant IFN-β could upregulate PGAM5 and PGAM5-mediated mitochondrial fission to rescue neurodegeneration in PD mouse models (Tresse et al., 2021). Along this line, IFN-β therapy has been applied to treat multiple sclerosis patients (Jakimovski et al., 2018). Thus, the underlying mechanism through affecting PGAM5 level and mitochondrial homeostasis could readily apply to a number of neurological diseases. Moreover, LFHP-1c inhibited phosphatase activity of PGAM5 and reduced the interaction between PGAM5 and NRF2 in rat brain microvascular endothelial cells. LFHP-1c treatment increased pDRP1(Ser637), but whether LFHP-1c represses mitochondrial fission is unclear (Gao et al., 2021). Since the phosphatase activity of PGAM5 is required for its participation in mitochondrial fission, mitophagy and mitochondrial biogenesis (Wang et al., 2012; Chen et al., 2014; Bernkopf et al., 2018), LFHP-1c might open up a new therapeutic strategy to regulate mitochondrial homeostasis. While several lines of evidence suggest the crucial role of mitochondrial function in neurological diseases, it raises a possibility that mitochondrial biogenesis may be the savior. In fact, impairment of mitochondrial biogenesis has been implicated in mitochondrial dysfunction in mouse PD model and the hippocampal tissues of AD patients (Sheng et al., 2012; Stevens et al., 2015). Accumulating evidence demonstrate that mitochondrial transplantation restored mitochondrial function and improved behaviors in PD mice and neuronal regeneration upon TBI (Chien et al., 2018; Chang et al., 2021). These reports showcase the beneficial effect of increasing mitochondrial mass in neurological diseases. PGAM5 enhanced mitochondrial biogenesis in liver and C2C12 myoblast cells (Bernkopf et al., 2018; Hong and Lee, 2018). Whether PGAM5 regulates mitochondrial biogenesis in neuronal cells is not known. Various approaches to regulate the level, activity, and cellular distribution of PGAM5 are suggested to examine the outcome on neurological diseases.

## Author Contributions

Conceptualization: LC and M-ZL. Writing and editing: M-ZL, LC and T-LK. Visualization: M-ZL and T-LK. Funding acquisition: LC. All authors contributed to the article and approved the submitted version.

## Conflict of Interest

The authors declare that the research was conducted in the absence of any commercial or financial relationships that could be construed as a potential conflict of interest.

## Publisher’s Note

All claims expressed in this article are solely those of the authors and do not necessarily represent those of their affiliated organizations, or those of the publisher, the editors and the reviewers. Any product that may be evaluated in this article, or claim that may be made by its manufacturer, is not guaranteed or endorsed by the publisher.

## Abbreviations

AD: Alzheimer’s disease
AREs: antioxidant response elements
ATP: adenosine triphosphate
CCCP: carbonyl cyanide m-chlorophenyl hydrazine
DRP1: dynamin-related protein 1
FIS1: mitochondrial fission 1 protein
FUNDC1: FUN14 Domain Containing 1
GTP: guanosine-5’-triphosphate
ICP34.5: infected-cell protein 34.5
IFN-β: interferon-β
KEAP1: Kelch-like ECH-associated protein 1
MAPK: mitogen activated protein kinase
MFN1: mitofusin 1
MFN2: mitofusin 2
MIM: mitochondrial inner membrane
Miro1: mitochondrial Rho GTPase 1
Miro2: mitochondrial Rho GTPase 2
MOM: mitochondrial outer membrane
mtDNA: mitochondrial DNA
NRF1: nuclear respiratory factor 1
NRF2: nuclear respiratory factor 2
OPA1: optic atrophy 1
PARL: presenilin-associated rhomboid-like
PD: Parkinson’s disease
PGAM: phosphoglycerate mutase
PGC1-α: peroxisome proliferator-activated receptor-γ coactivator 1-α
PINK1: phosphatase and tensin homolog-induced putative kinase protein 1
ROS: reactive oxygen species
STAT5: signal transducer and activator of transcription 5
TBI: traumatic brain injury
TFAM: mitochondrial transcription factor A.
